# Use and Misuse of Nitrogen in Agriculture: The German Story

**DOI:** 10.1100/tsw.2001.263

**Published:** 2001-10-30

**Authors:** Rienk R. van der Ploeg, Rienk P. Schweigert, Rienk J. Bachmann

**Affiliations:** Institute of Soil Science, University of Hannover, Germany

**Keywords:** nitrogen, agriculture, environment, crop production, animal production, fertilizers, fodder, theory of mineral nutrition of plants, Carl Sprengel, Justus von Liebig, history of agronomy, nitrate, ammonia, nitrous oxide, groundwater contamination, trace gas emissions, climate change, damage to human health, obesity, adipositas, external costs of agriculture

## Abstract

Nitrogen (N) fertilization in agriculture has been discussed controversially in Germany for almost two centuries. The agronomist Carl Sprengel, who published his theory on the mineral nutrition of plants in 1828, advocated the use of mineral N fertilizers. Chemist Justus von Liebig, on the other hand, vehemently denied around 1850 the need for N fertilization. Although it soon became evident that Sprengel was right and Liebig was wrong, not much synthetic N fertilizer was used in German agriculture until around 1915, when the Haber-Bosch technique enabled the commercial production of NH_3_. The use of N fertilizers since then has grown, especially since 1950. To increase agricultural productivity, German governments have promoted, directly and indirectly, the use of N in crop and in animal production. Unfortunately, it was overlooked that N surpluses in agriculture increased rapidly; around 1980 they amounted yearly to more than 100 kg ha. The extensive use of N in agriculture is causing environmental damage and is contributing substantially to the external costs of present agriculture. The main N compounds that affect the environment are N_2_O, NH_3_, and NO_3_. These compounds are considered to contribute one third to the external costs of agriculture. Additionally, the high rate of human intake of animal proteins and lipids has adversely affected the health of the country’s population. Fundamental corrections in German farm policy appear inevitable.

## INTRODUCTION

The Swiss botanist Théodore de Saussure (1765–1845) is generally considered to be the founder of (experimental) plant physiology. Among his many findings is the discovery (published in 1804) that plants draw their nitrogen (N) from the soil on which they grow[1], but not from the air. Initially, though, his work did not receive much attention[2]. The German agronomist and chemist Carl Sprengel (1787–1859), who was familiar with the work of de Saussure, was possibly the first scholar who recommended the use of mineral fertilizers for field crops. Sprengel, one of the founders of agricultural chemistry, carried out epoch-making agronomic research at the University of Göttingen from 1821 to 1831[3]. In 1826 he disproved the so-called humus theory[4], and in 1828 he published his theory on the mineral nutrition of plants[5]. Besides the inherent elements C, H, O, and Si, Sprengel considered N, Ca, K, Na, Mg, P, and S as well as Fe, Mn, Cl, and Al as essential plant constituents. He listed also Cu as a possible plant nutrient. The list of Sprengel[5] thus contained 10 (N, Ca, K, Mg, P, S, Fe, Mn, Cl, and Cu) of the 13 elements that today are considered essential plant nutrients.

Sprengel was probably the first agronomist who conducted controlled experiments with mineral fertilizers. As early as 1828, he recommended the use of $NH_3$ and $NO_3$ as fertilizers. In his pioneering 1828 publication, he discussed not only the fertilizing action of $(NH_4)NO_3$, $(NH_4)_2SO_4$, $(NH_4)Cl$, and $(NH_4)_2CO_3$, but also of $KNO_3$, $NaNO_3$, and $Ca(NO_3)_2$. In this respect it is interesting to note that also in 1828, Friedrich Wöhler (1800–1882) appears to have produced the first synthetic urea ($CO[NH_2]_2$). However, it took nearly 100 years before urea was commercially produced (see the next section). Wöhler, like Sprengel, worked at the University of Göttingen and is further known because of his discovery of the chemical elements Si and Al, among others.

Figure 1 shows an undated photograph of Carl Sprengel. Sprengel was a highly productive and respected agronomist in Germany during the first half of the 19th century. Besides several journal articles, he authored a number of textbooks, such as on agricultural chemistry and on fertilizer use[6,7,8]. Because all his work was published in German, Sprengel received little international recognition.

Around 1850 the chemist Justus von Liebig (1803–1873) vehemently denied the necessity of fertilizing field crops with N. According to Liebig (Carl Sprengel's opponent for many years), field crops obtain all the N they need from the large $NH_3$ pool of the atmosphere, of which part is carried into the soil by precipitation. His dispute with the British agronomist John Lawes (1814–1900) of Rothamsted on this subject especially received wide attention and lasted for more than 10 years[9,10]. Because Liebig did not have a background in agronomy, he advocated, particularly in the early part of his agronomic career, occasionally erroneous views. More serious, however, was the ethical misconduct that Liebig showed when he entered the field of agricultural chemistry[11] in 1840. Drawing extensively on the work of Carl Sprengel, Liebig passed on Sprengel's doctrines on the mineral nutrition of plants as his own, ignoring insolently the reproaches of Sprengel and others about originality and priority. Liebig's reputation as the father of modern agriculture is therefore blemished by (uncastigated) plagiarism.

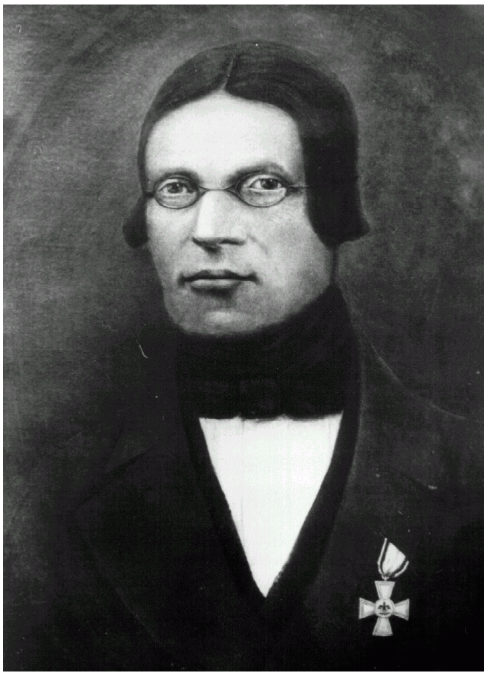

**FIGURE 1.** Undated photograph of the agronomist and chemist Carl Sprengel (1787–1859), one of the founders of agricultural chemistry, who recommended the use of mineral N fertilizers as early as 1828.

Although it soon became obvious that Liebig's views on fertilizer N were wrong and those of others, such as Sprengel and Lawes, were correct, not much mineral (commercial) N fertilizer was used in Germany in the 19th century, simply because it was not available. Some $NaNO_3$ from Chile and some guano from Peru were imported, but the use of these N fertilizers was never widespread because of high transportation costs. Also the use of $(NH_4)_2SO_4$, a byproduct from coke production, never reached a high level. Until the end of the 19th century, Germany, like most other countries, depended mainly on noncommercial sources of N for its agriculture[12,13]. These included farm manure, legumes, and human wastes. It is estimated[14] that the average use of fertilizer N in German agriculture in 1900 was 22.7 kg $ha^{-1}$ $year^{-1}$, of which 20.5 kg $ha^{-1}$ was farm manure and only 2.2 kg $ha^{-1}$ commercial N fertilizer.

## NITROGEN USE IN GERMAN AGRICULTURE

Figure 2 illustrates the use of commercial N fertilizers in Germany. The first N fertilizers were produced in Germany in the early 20th century. The production of calcium cyanamide ($CaCN_2$) started in 1905 at Westeregeln, near Magdeburg, in northern Germany. The so-called Frank-Caro procedure was named after the chemists Adolf Frank (1834–1916) and Nikodem Caro (1871–1935). Because the production required large amounts of electrical energy, fertilizer production was costly and the product applied only moderately in agriculture[13,15].

A less costly commercial N fertilizer was produced only a few years later. Around 1910, the chemist Fritz Haber (1868–1934) successfully synthesized $NH_3$ by a catalytic reaction of N with H. Together with Carl Bosch (1874–1940), he developed a procedure to manufacture $NH_3$ commercially[13] — in 1913 at Oppau/Ludwigshafen in southern Germany (BASF Company). For their work, Haber was awarded the Nobel Prize in 1918 and Bosch in 1931. Also important for agriculture was a procedure developed around 1925 by BASF and the I.G. Farben Company in Frankfurt that led to commercial urea manufacturing. However, it was not until after the end of World War II that this fertilizer was applied at a large scale to agriculture in Germany and other parts of the world.

Today other commercial N fertilizers are used as well in German agriculture[14,15,16], and their use now has reached a level that raises environmental concerns. Figure 2 shows the increasing use of commercial N fertilizer in German agriculture. Until 1950 the use changed rather slowly, but since then a dramatic increase is observed. Since the reunification of East and West Germany in 1990, the rate of commercial N fertilizer use has slowed somewhat, but still amounts to more than 100 kg $ha^{-1}$ $year^{-1}$ of farmland.

Commercial fertilizer is not the only (external) source of N in German agriculture. Germany has, since the 1950s, increasingly imported protein-rich fodder[14,17] for its rapidly growing livestock population. A maximum equivalent rate of N use of 55.2 kg $ha^{-1}$ $year^{-1}$ was reached in the 1980s[17]. Since then, the use of imported fodder has decreased to about 25 kg $ha^{-1}$ $year^{-1}$, but this source of N adds still considerably to the yearly N turnover in German agriculture.

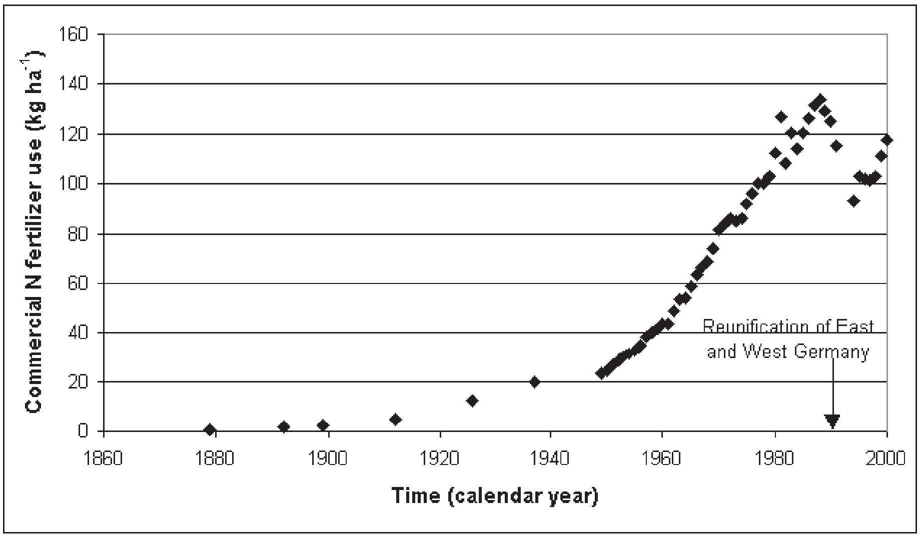

**FIGURE 2.** Use of commercial N fertilizer (in kg ha$^{-1}$ of farmland) in Germany from 1860 to 2000[14].

## NITROGEN SURPLUSES IN GERMAN AGRICULTURE AND ENVIRONMENTAL CONCERNS

Federal German governments have succeeded in substantially raising the country's postwar agricultural productivity. Although the population and consumption of agricultural products such as meat, eggs, and dairy products grew steadily at the same time as the area of agricultural land decreased[14,17], Germany has become self-sufficient in many areas of agricultural production. Figure 3 shows the degree of self-sufficiency for wheat, rye, beef, and sugar in the postwar era. There is no doubt that the increased use of N in agriculture has contributed significantly to the increased agricultural productivity achieved after World War II.

However, the increased use of N in agriculture had negative effects as well. The environment especially, but also indirectly the public health, suffered from the high rate of N use in agriculture. In a glimpse of the environmental implications, Figure 4 shows the N input (commercial fertilizer plus imported livestock fodder) in German agriculture, as well as the N output (in meat, flour, eggs, milk, etc.) per year. The difference between both curves represents the yearly N surplus (in kg ha$^{-1}$) with which German agriculture is operating. Notice that N inputs from atmospheric deposition, sludge application, or legume cultivation are not considered in Figure 4. The N surplus in German agriculture in recent years has decreased somewhat, but it still amounts to more than 80 kg ha$^{-1}$. In view of the total agricultural land area (in 1997 about 54% of the German territory), it is likely that the enormous yearly N turnover in agriculture affects the environ-

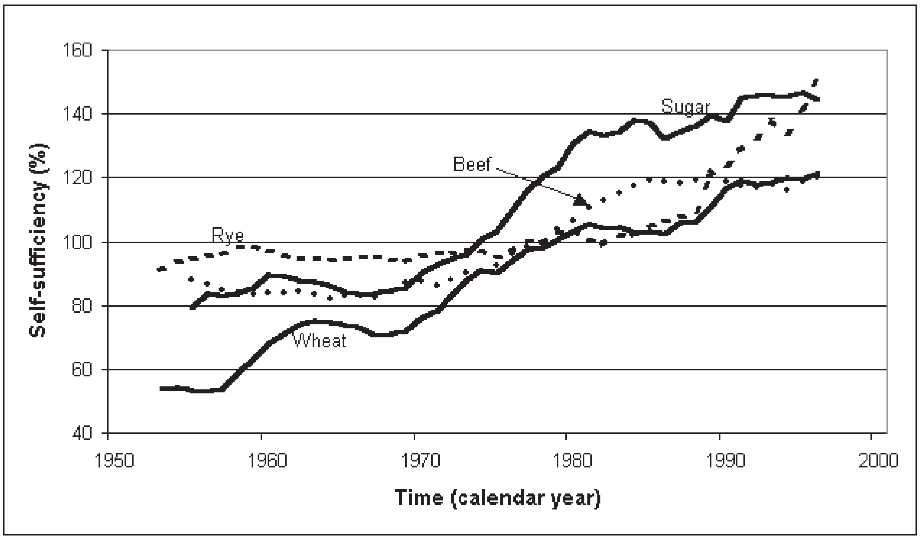

**FIGURE 3.** Postwar German self-sufficiency for wheat, rye, beef, and sugar[14].

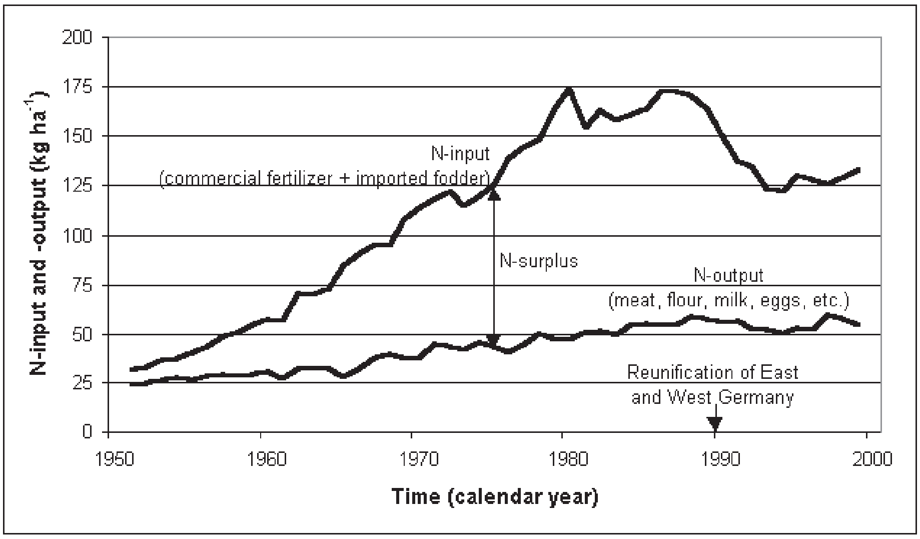

**FIGURE 4.** Nitrogen surplus (in kg ha$^{-1}$ of farmland) in German agriculture between 1950 and 2000[17].

ment. This applies particularly to the country's natural resources of water and air. Contamination of these resources with the agriculturally produced chemical compounds $NO_3$, $NH_3$, and $N_2O$ is today recognized as a major environmental problem[18,19,20, 21,22].

To illustrate the $NO_3$ problem, data from the state of Niedersachsen (Lower Saxony) in northern Germany can be presented. To learn the extent of the agriculturally caused $NO_3$ problem, Lower Saxony carried out a statewide soil $NO_3$ assessment program in the fall of 1985 to 1987. A regular grid with a node distance of 12 km was laid on top of the state (Figure 5) and soil samples (from the 0 to 30, 30 to 60, and 60 to 90 cm depth) were taken 3 years in a row at the nodes, regardless of the local land use. For each of the three depths, soil $NO_3$-N was determined[23]. The results of this program are given in Table 1. The average $NO_3$-N value for cropland in the period from 1985 to 1987 was 75 kg ha$^{-1}$ and for grassland, 50 kg ha$^{-1}$. The time of seepage in Lower Saxony is roughly the winter period (November-April), and the average rate of seepage is about 200 mm year$^{-1}$[24]. If it is assumed that all the fall $NO_3$ is leached from the soil during the winter months, then the average $NO_3$ concentration in the seepage under cropland during 1985 to 1987 was 165 mg l$^{-1}$ and under grassland, 110 mg l$^{-1}$. Because Germany depends heavily on groundwater for its drinking water supply, and because the (upper) limit for the $NO_3$ concentration in drinking water since 1986 is 50 mg l$^{-1}$ (or 11.4 mg l$^{-1}$ $NO_3$-N), it is apparent that Germany has a $NO_3$ problem from the extensive use of N in its agriculture.

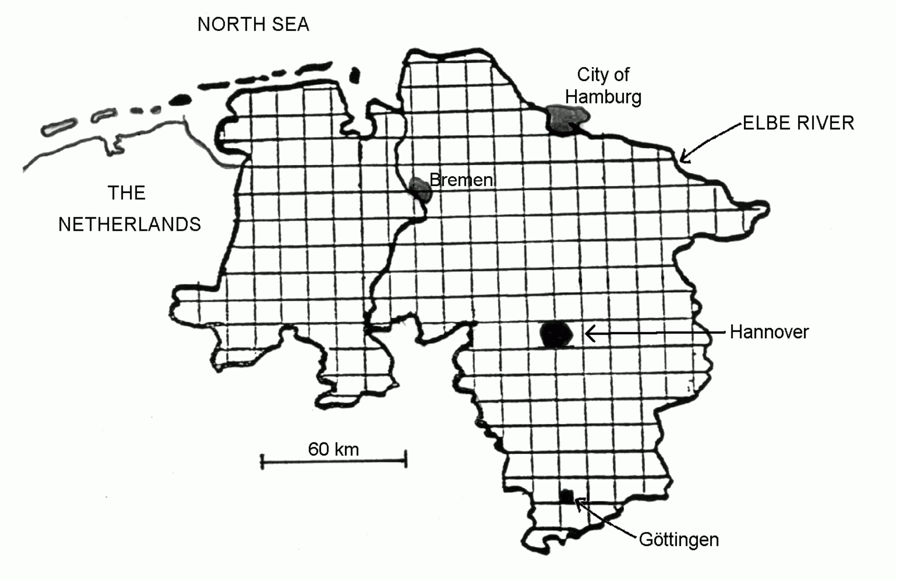

**FIGURE 5.** Map of the state of Niedersachsen (Lower Saxony) in northern Germany, covered with a soil nitrogen assessment grid as used in the period from 1985 to 1987.

**TABLE 1**
**Distribution and Total Amount of Soil Mineral N in Early November in the State of Niedersachsen (Lower Saxony, Germany) in the Period from 1985 to 1987[23]**

| | Soil depth | | | |
|---|---|---|---|---|
| **Land use** | **0 – 30 cm** | **30 – 60 cm** | **60 – 90 cm** | **0 – 90 cm** |
| | **kg ha$^{-1}$ $NO_3$-N** | | | |
| Cropland, n = 450 | 32 | 24 | 19 | 75 |
| Grassland, n = 226 | 25 | 13 | 12 | 50 |
| Woodland, n = 131 | 20 | 8 | 6 | 34 |
| Wasteland, n = 42 | 20 | 5 | 4 | 29 |
| Preserve, n = 5 | 2 | 2 | 3 | 7 |
| Nursery, n = 3 | 140 | 83 | 62 | 285 |
| Vegetable-cropland, n = 6 | 19 | 66 | 47 | 132 |
| Orchard, n = 6 | 25 | 7 | 5 | 37 |

German agriculture is also contributing substantially to the emission of the trace gases $NH_3$ and $N_2O$. For 1990, for example, the European Centre for Ecotoxicology and Toxicology of Chemicals[19] estimated the $NH_3$-N emission in (former) West Germany at 664 Gg. Nearly 92% of this amount was attributed to agriculture, which corresponds to a $NH_3$-N emission of 48 kg ha$^{-1}$. A rough estimate about the $N_2O$ emission due to German agriculture can also be made. To this end, a model proposed by Bouwman[25] can be used. This model estimates the yearly $N_2O$-N emission (in kg ha$^{-1}$) from the yearly amount of applied N fertilizer (in kg ha$^{-1}$). In 1996, for example, the average amount of applied N fertilizer in Germany was 177.4 kg ha$^{-1}$ (102 kg ha$^{-1}$ commercial fertilizer plus 75.4 kg ha$^{-1}$ manure). With the Bouwman model[25], a $N_2O$-N emission of 3.22 kg ha$^{-1}$ is calculated. Thus an $N_2O$-N emission of 56 Gg is estimated for the total area of German farmland in 1996 (17.3 Mha). This value compares with the $N_2O$-N emission of agriculture in 1996 in the U.K., given by Pretty et al.[26] as 62 Gg.

## EXTERNAL COSTS OF AGRICULTURE

An attempt was made recently to assess the total external costs of agriculture in the U.K.[26]. The authors of this study distinguished between costs due to damage to the natural capital (water, air, soil, biodiversity/landscape) and damage to human health (pesticides, nitrate, microorganisms/other disease agents). For 1996 they estimated the total costs in the U.K. at £2343 million, or at £208 ha$^{-1}$ (˜US$300 ha$^{-1}$). The costs from $NO_3$ in sources of drinking water were estimated at £16 million, because of $NH_3$ emissions at £48 million, and because of $N_2O$ emissions at £738 million. This means that the costs due to the release of reactive agricultural N compounds into the environment were estimated at about one third of the total external costs, with $N_2O$ considered the most damaging component. The marginal costs of the air pollutants $NH_3$ and $N_2O$ were estimated at £171 Mg$^{-1}$ and £7530 Mg$^{-1}$, respectively.

To date, a detailed study has not been made to determine the external costs of German agriculture. If, for convenience, it is assumed that the agricultural conditions in Germany are similar to those in the U.K., the yearly damage to the environment in Germany can also roughly be estimated. In 1996, the total area of farmland in Germany was 17.3 million ha[14]. If it is assumed that the external costs per hectare of German farmland in 1996 were the same as in the U.K. (˜US$300), total costs of about U.S.$5000 million are calculated. If, as in the U.K., one third of these costs can be attributed to $NO_3$, $NH_3$, and $N_2O$, the total environmental costs in 1996 in Germany due to excessive use of N in agriculture were US$1700 million.

It is interesting to compare these values (U.S.$5000 million for the total external costs and U.S.$1700 million for the costs caused by the reactive N compounds) with the net worth of all farm commodities produced in the country in 1996. According to the 1996 Statistical Yearbook of Agriculture (Table 182)[14], this net worth was DM 23,100 million (˜US$11,600 million). If, however, the European Common Market price guarantees for some of these commodities taken into account and if the corresponding subsidies are subtracted, the net worth was only DM 13,000 million (˜U.S.$6500 million). The comparison indicates that the external costs, caused yearly by agriculture in Germany, presently correspond to the total worth of all farm goods produced. Abundant use of N is considered to contribute about one third to these external costs.

## NITROGEN USE IN AGRICULTURE AND ITS AFFECT ON PUBLIC HEALTH

Some 20 years ago there was much concern in Germany and other countries in Western Europe about rising $NO_3$ concentrations in the groundwater. Groundwater is by far the main source of potable water in Germany (˜75%). Nitrate was considered to be a potential health hazard, associated with stomach cancer and the so-called blue-baby syndrome (methaemoglobinaemia)[18].

Although the groundwater quality in recent years barely improved, water companies appear to have developed efficient ways to remove $NO_3$ from their water. In the aforementioned study about the external costs of U.K. agriculture[26], the human health costs by $NO_3$ were considered negligible, but those caused by removing $NO_3$ from raw potable water were set at £16 million. Hence, immediate health problems due to food with elevated amounts of $NO_3$ rarely occur.

Indirectly, the ample use of N in agriculture did affect the public health. The German diet changed simultaneously with an increased use of N in agriculture. Figure 6 shows the increased consumption of meat between 1950 and 2000. Whereas in the early 1950s meat consumption was only 40 kg year$^{-1}$ per capita, in the 1980s it peaked at more than 100 kg year$^{-1}$. Since then the meat consumption has gone down somewhat; today it is nearly 95 kg year$^{-1}$. The consumption of dairy products and eggs shows a similar development[17].

Associated with an increased consumption of meat and other animal products, the average diet became increasingly rich in energy. Figure 7 shows the daily amount of protein consumed between 1951 and 1987. Also shown is the fraction of animal protein. Whereas the amount of total protein changed from 78 g per capita in 1951 to 94 g in 1987, the percentage of animal protein increased from 48 to 68%. For comparison it is noted that the U.S. recommended daily allowance (RDA) for dietary protein is 60 g and that the WHO recommendation is 0.75 g kg$^{-1}$ of body weight[27]. A similar development as for protein is observed for fat; in 1951 the daily fat consumption per capita was 102 g, but by 1987 it had increased to 161 g. At the same time, the daily intake of food energy increased from 2867 kcal per capita to 3431 kcal. (The latter value excludes the energy intake with alcohol, which constitutes another 255 kcal per capita daily.) The energy fraction derived from animal products (such as fat) in 1951 was 29%, but had risen to 41% in 1987.

Diets containing more than 25% of all food energy from animal fat are considered to be a main cause of obesity and of comorbidities, such as diabetes, stroke, coronary heart disease, and different cancers[27]. Obesity is usually expressed in terms of the Body Mass Index (BMI)[28]. This index (kg m$^{-2}$) represents the ratio of a person's weight (kg) and his height (m) squared. A BMI exceeding 25 denotes overweight. If a person's BMI is 30 or higher, he is considered to suffer from adiposity, a chronic obesity disease. In Germany it is estimated that presently 60% of all adults are overweight, and that almost 20% suffer from adiposity[29]. The public health costs in Germany associated with overweight and obesity are exorbitant[30]; they are a multiple of the external costs of agriculture. It should be realized that these costs are also related to agriculture and to the excessive use of N.

## GOVERNMENT POLICIES REGARDING NITROGEN USE IN AGRICULTURE

It is increasingly recognized in Germany that the conventional postwar farm policy may need reconsideration. Although there is an overproduction of milk, beef, sugar, and cereal, farmers are encouraged (by means of subsidies) to further increase their production. Yet, because agricultural commodities are priced low on the world market, it is often not possible to export German farm products unless they are resubsidized. An increased farm production, however, requires a higher input of fertilizers, pesticides, and fodder, hence further imposing on the environment. Besides environmental anxieties, there is also an increasing public concern about food quality (triggered again recently by the BSE epidemic), about mass animal husbandry, and about the excessive public funds (subsidies) granted to agriculture (almost 50% of the EU budget). Therefore, voices that demand farm policy changes are becoming louder.

The present government coalition of Socialist-Democrats and Environmentalists in Germany is working out plans to reform conventional agriculture. Tax levies on N fertilizer, on pesticide use, on imported fodder, and on mass animal husbandry are being considered[31] as part of a comprehensive so-called ecological tax reform. So far, though, only minor changes in farm

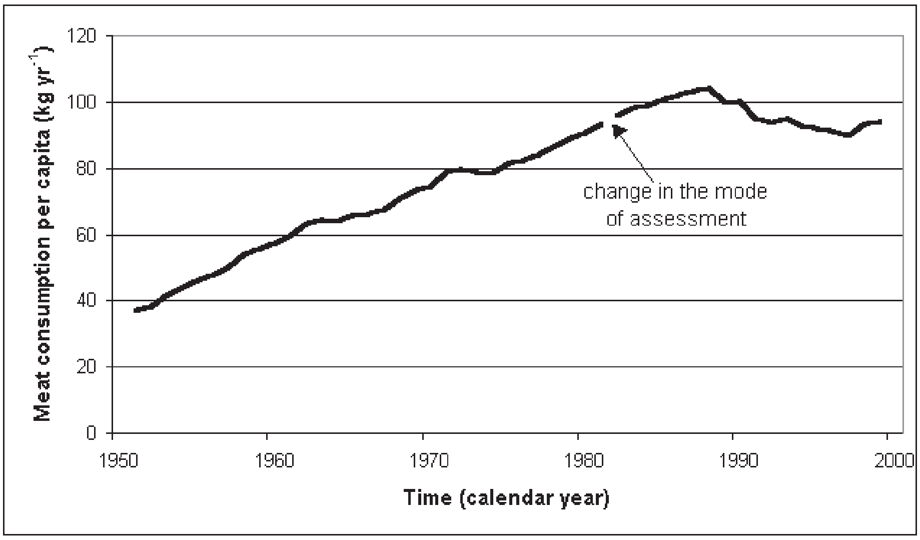

**FIGURE 6.** Postwar meat consumption (in kg year$^{-1}$ per capita) in Germany; in the mid-1980s there was a change in the assessment mode [14].

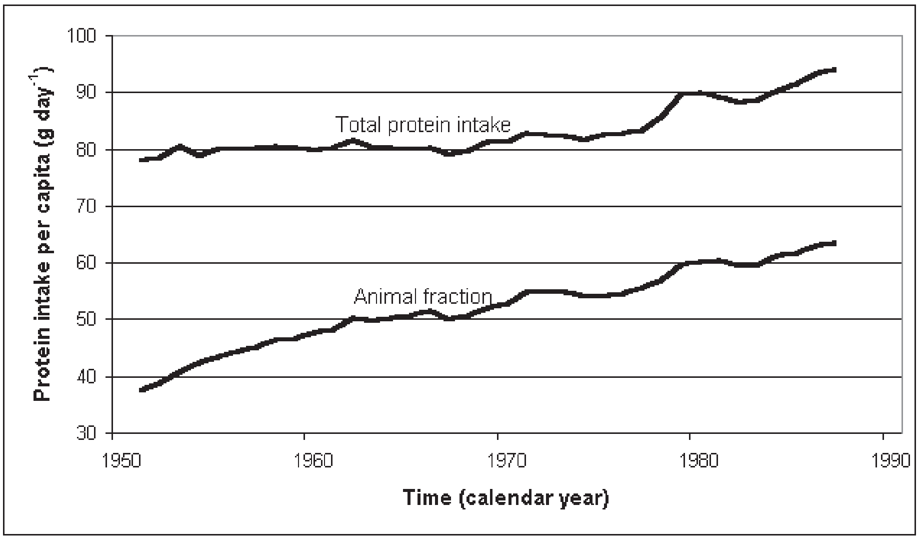

**FIGURE 7.** The daily total protein intake per capita in postwar Germany and the increase of the animal fraction in the course of time[14].

policy have been realized. Furthermore, opponents of the government plans question the effectiveness of the anticipated tax levies. They argue that unless such measures are imposed globally, they hardly relieve the environment, but instead encumber national agriculture. It seems indeed that only global or multilateral agreements will help reduce the external costs that agriculture with extensive use of N is causing. Recent experience in other areas, e.g., the reduction of $CO_2$ emissions, shows that we have a long way to go before such agreements can be reached.

## CONCLUSION

The use of N fertilizers in Germany during the past century has helped raise its agricultural productivity and prosperity. Meanwhile, however, N use has reached a level significantly affecting environment and the public health. Nitrous oxide emission especially causes great concern. The excessive use of N fertilizers, in Germany as well as in other parts of the world, needs to be reconsidered. To avoid international economic dislocations, global or multilateral agreements on reduced use of N in agriculture appear to be desirable.

| | | |
|---|---|---|
| Received: | July | 27, 2001 |
| Revised: | September | 20, 2001 |
| Accepted: | October | 3, 2001 |
| Published: | October | 30, 2001 |
